# 155 Impact of a Hand Hygiene Education Class Targeting Onboarding Dietary Staff on Hand Hygiene Compliance Rates

**DOI:** 10.1017/ash.2026.10556

**Published:** 2026-06-23

**Authors:** Thang Phung Manh, Duy Nguyen Xuan Nhat, An Nguyen Thai, Nhieu Nguyen Van, Sy Hoang Van

**Affiliations:** 1 Cho Ray Hospital

## Abstract

**Background:** Central line-associated bloodstream infection (CLABSI) is one of the four most common healthcare-associated infections worldwide and often results in serious consequences, including prolonged treatment duration, increased antibiotic usage costs, and higher mortality risk. In Vietnam, the CLABSI rate in intensive care units (ICUs) remains high, ranging from 5 to 10 episodes per 1,000 central venous catheter (CVC)-days. Therefore, implementing a CLABSI prevention bundle in ICUs is urgently necessary to ensure patient safety. **Methods:** A CLABSI prevention bundle was developed, consisting of an insertion bundle and a maintenance care bundle. Training and implementation were conducted in three ICU units: Neurosurgical ICU Dept. (NsICU), Internal Cardiology Dept. (IC), and Cardiac surgery resuscitation Dept. (CSR) from February to September 2025. External audits were conducted on all CVC insertion cases and 5% of CVC maintenance care opportunities in the three departments. **Results:** The total number of insertion bundle observation opportunities in NsICU, IC, and CSR were 67, 35, and 43 respectively, with average compliance rates increasing over time as follows: 96.76% (range 91.67–100%), 89.46% (range 79.63–95.83%), and 97.18% (range 85–100%). The total number of maintenance care observation opportunities in NsICU, IC, and CSR were 86, 63, and 53 respectively, with average compliance rates over time increasing as follows: 78.07% (range 70–87.5%), 87.75% (range 75–100%), and 82.41% (range 72.73–100%). Despite this, there were certain periods with low compliance rates for both the insertion and maintenance care bundles, particularly in areas such as proper hand hygiene procedures, insufficient antiseptic contact time, and non-compliance with drying time requirements. The average CLABSI rates post-intervention compared to pre-intervention in NsICU, IC, and CSR decreased from 2.38 to 1.6 (P=0.54), from 3.15 to 2.57 (P=0.68), and from 2.4 to 2.02 (P=0.75), respectively. **Conclusion:** The initial implementation of the CLABSI prevention bundle in three clinical departments led to progressively improved compliance with both CVC insertion and maintenance care practices, contributing to a reduction in CLABSI rates compared to the pre-intervention period.

